# Supplementary material for: Modeling glioblastoma heterogeneity as a dynamic network of cell states
Source: Mol Syst Biol. 2021 Sep 16;17(9):e10105. doi: 10.15252/msb.202010105 (PMC8444284; doi:10.15252/msb.202010105)
Supplement: Supplementary file 6 — Source Data for Figure 5 [file MSB-17-e10105-s004.zip › Figure5A_sourcedata/GSEA_3071/nosigdb_stateII.GseaPreranked.1622795151171/RICHARDS_NOWAKOWSKI_SCIENCE_2017_ASTROCYTE_UPREG.html]

Details for gene set RICHARDS\_NOWAKOWSKI\_SCIENCE\_2017\_ASTROCYTE\_UPREG[GSEA]

|  || Dataset | state23071 |
| Phenotype | NoPhenotypeAvailable |
| Upregulated in class | na\_neg |
| GeneSet | RICHARDS\_NOWAKOWSKI\_SCIENCE\_2017\_ASTROCYTE\_UPREG |
| Enrichment Score (ES) | -0.5346133 |
| Normalized Enrichment Score (NES) | -3.5150385 |
| Nominal p-value | 0.0 |
| FDR q-value | 0.0 |
| FWER p-Value | 0.0 |
Table: GSEA Results Summary

  

Fig 1: Enrichment plot: RICHARDS\_NOWAKOWSKI\_SCIENCE\_2017\_ASTROCYTE\_UPREG      
 Profile of the Running ES Score & Positions of GeneSet Members on the Rank Ordered List

  

| PROBE | GENE SYMBOL | GENE\_TITLE | RANK IN GENE LIST | RANK METRIC SCORE | RUNNING ES | CORE ENRICHMENT || 1 | F3 |  |  | 13 | 1.555 | 0.0042 | No |
| 2 | MT1E |  |  | 106 | 1.033 | -0.0149 | No |
| 3 | AXL |  |  | 129 | 0.997 | -0.0158 | No |
| 4 | ADAMTS6 |  |  | 191 | 0.884 | -0.0275 | No |
| 5 | EGFR |  |  | 198 | 0.874 | -0.0248 | No |
| 6 | DLC1 |  |  | 208 | 0.868 | -0.0229 | No |
| 7 | CYR61 |  |  | 267 | 0.791 | -0.0343 | No |
| 8 | ALCAM |  |  | 288 | 0.771 | -0.0358 | No |
| 9 | DGKH |  |  | 301 | 0.754 | -0.0353 | No |
| 10 | MT2A |  |  | 393 | 0.682 | -0.0558 | No |
| 11 | PHGDH |  |  | 429 | 0.664 | -0.0618 | No |
| 12 | ZFP36L2 |  |  | 458 | 0.647 | -0.0659 | No |
| 13 | ENO1 |  |  | 501 | 0.629 | -0.0739 | No |
| 14 | PDLIM5 |  |  | 526 | 0.613 | -0.0772 | No |
| 15 | TACC1 |  |  | 534 | 0.610 | -0.0761 | No |
| 16 | SSR3 |  |  | 557 | 0.598 | -0.0789 | No |
| 17 | MEST |  |  | 563 | 0.593 | -0.0773 | No |
| 18 | SCCPDH |  |  | 582 | 0.586 | -0.0792 | No |
| 19 | NTM |  |  | 592 | 0.582 | -0.0787 | No |
| 20 | RGCC |  |  | 600 | 0.578 | -0.0777 | No |
| 21 | EPB41L2 |  |  | 623 | 0.569 | -0.0807 | No |
| 22 | SLC25A5 |  |  | 680 | 0.537 | -0.0928 | No |
| 23 | PNPT1 |  |  | 728 | 0.523 | -0.1026 | No |
| 24 | PDXK |  |  | 752 | 0.515 | -0.1061 | No |
| 25 | SNX5 |  |  | 762 | 0.512 | -0.1060 | No |
| 26 | HOPX |  |  | 768 | 0.510 | -0.1048 | No |
| 27 | ID1 |  |  | 769 | 0.509 | -0.1023 | No |
| 28 | SLC2A1 |  |  | 787 | 0.503 | -0.1043 | No |
| 29 | LRP1 |  |  | 856 | 0.483 | -0.1198 | No |
| 30 | RGS20 |  |  | 942 | 0.461 | -0.1399 | No |
| 31 | SLC7A11 |  |  | 998 | 0.445 | -0.1521 | No |
| 32 | DDAH1 |  |  | 1028 | 0.434 | -0.1576 | No |
| 33 | NOG |  |  | 1031 | 0.433 | -0.1560 | No |
| 34 | FGF1 |  |  | 1058 | 0.426 | -0.1608 | No |
| 35 | SSPN |  |  | 1081 | 0.421 | -0.1645 | No |
| 36 | EFHD2 |  |  | 1090 | 0.419 | -0.1645 | No |
| 37 | OGFRL1 |  |  | 1099 | 0.417 | -0.1646 | No |
| 38 | NOTCH3 |  |  | 1141 | 0.409 | -0.1734 | No |
| 39 | COL11A1 |  |  | 1163 | 0.404 | -0.1769 | No |
| 40 | SMC5 |  |  | 1181 | 0.401 | -0.1794 | No |
| 41 | APCDD1 |  |  | 1238 | 0.389 | -0.1922 | No |
| 42 | HSDL2 |  |  | 1326 | 0.373 | -0.2132 | No |
| 43 | PCDH7 |  |  | 1329 | 0.373 | -0.2119 | No |
| 44 | GLUD1 |  |  | 1397 | 0.358 | -0.2278 | No |
| 45 | HSPA1A |  |  | 1409 | 0.356 | -0.2289 | No |
| 46 | PSAT1 |  |  | 1505 | 0.340 | -0.2522 | No |
| 47 | THG1L |  |  | 1535 | 0.334 | -0.2582 | No |
| 48 | PHACTR2 |  |  | 1596 | 0.324 | -0.2723 | No |
| 49 | COL6A1 |  |  | 1633 | 0.319 | -0.2802 | No |
| 50 | FAM120A |  |  | 1642 | 0.318 | -0.2808 | No |
| 51 | HSPA2 |  |  | 1688 | 0.312 | -0.2911 | No |
| 52 | JUN |  |  | 1725 | 0.307 | -0.2990 | No |
| 53 | SWAP70 |  |  | 1751 | 0.304 | -0.3041 | No |
| 54 | PRTFDC1 |  |  | 1866 | 0.290 | -0.3326 | No |
| 55 | WWC2 |  |  | 1901 | 0.285 | -0.3401 | No |
| 56 | SDC3 |  |  | 1908 | 0.284 | -0.3403 | No |
| 57 | TXNIP |  |  | 1918 | 0.283 | -0.3413 | No |
| 58 | CPT1A |  |  | 1959 | 0.278 | -0.3505 | No |
| 59 | FERMT2 |  |  | 2003 | 0.272 | -0.3604 | No |
| 60 | RCN1 |  |  | 2051 | 0.267 | -0.3715 | No |
| 61 | UGP2 |  |  | 2141 | 0.258 | -0.3936 | No |
| 62 | SERPINH1 |  |  | 2146 | 0.258 | -0.3933 | No |
| 63 | ANXA5 |  |  | 2187 | 0.253 | -0.4026 | No |
| 64 | LINC01138 |  |  | 2189 | 0.253 | -0.4016 | No |
| 65 | PKM |  |  | 2199 | 0.252 | -0.4028 | No |
| 66 | PEA15 |  |  | 2209 | 0.251 | -0.4039 | No |
| 67 | GPC6 |  |  | 2232 | -0.251 | -0.4085 | No |
| 68 | ZIC1 |  |  | 2259 | -0.255 | -0.4140 | No |
| 69 | ZNF621 |  |  | 2270 | -0.256 | -0.4154 | No |
| 70 | NRBP2 |  |  | 2271 | -0.256 | -0.4142 | No |
| 71 | ATP9B |  |  | 2276 | -0.256 | -0.4140 | No |
| 72 | LRRTM3 |  |  | 2282 | -0.257 | -0.4140 | No |
| 73 | LRP4 |  |  | 2300 | -0.259 | -0.4172 | No |
| 74 | FADS2 |  |  | 2338 | -0.263 | -0.4256 | No |
| 75 | GJA1 |  |  | 2366 | -0.266 | -0.4314 | No |
| 76 | AGPAT3 |  |  | 2375 | -0.267 | -0.4322 | No |
| 77 | RAB13 |  |  | 2381 | -0.268 | -0.4322 | No |
| 78 | PSENEN |  |  | 2387 | -0.269 | -0.4322 | No |
| 79 | SEMA6D |  |  | 2407 | -0.272 | -0.4359 | No |
| 80 | NEK6 |  |  | 2411 | -0.272 | -0.4353 | No |
| 81 | DTNA |  |  | 2414 | -0.272 | -0.4345 | No |
| 82 | CANX |  |  | 2427 | -0.274 | -0.4363 | No |
| 83 | SNAP23 |  |  | 2452 | -0.277 | -0.4413 | No |
| 84 | OLIG2 |  |  | 2453 | -0.278 | -0.4399 | No |
| 85 | SPRY2 |  |  | 2455 | -0.278 | -0.4388 | No |
| 86 | CRYL1 |  |  | 2460 | -0.279 | -0.4385 | No |
| 87 | SRPK2 |  |  | 2475 | -0.280 | -0.4408 | No |
| 88 | ILDR2 |  |  | 2478 | -0.280 | -0.4400 | No |
| 89 | SEPT2 |  |  | 2482 | -0.281 | -0.4394 | No |
| 90 | HADHB |  |  | 2489 | -0.283 | -0.4396 | No |
| 91 | GATM |  |  | 2501 | -0.285 | -0.4411 | No |
| 92 | RORA |  |  | 2513 | -0.287 | -0.4426 | No |
| 93 | MLLT6 |  |  | 2573 | -0.297 | -0.4566 | No |
| 94 | ABI1 |  |  | 2598 | -0.301 | -0.4614 | No |
| 95 | DIO2 |  |  | 2599 | -0.301 | -0.4600 | No |
| 96 | HS2ST1 |  |  | 2620 | -0.304 | -0.4637 | No |
| 97 | TMEM132B |  |  | 2623 | -0.304 | -0.4628 | No |
| 98 | SRGAP2B |  |  | 2638 | -0.307 | -0.4650 | No |
| 99 | HSP90B1 |  |  | 2648 | -0.308 | -0.4658 | No |
| 100 | MFGE8 |  |  | 2712 | -0.319 | -0.4808 | No |
| 101 | FAM84B |  |  | 2713 | -0.319 | -0.4792 | No |
| 102 | IVD |  |  | 2723 | -0.321 | -0.4800 | No |
| 103 | DPM3 |  |  | 2738 | -0.324 | -0.4821 | No |
| 104 | PARP9 |  |  | 2780 | -0.332 | -0.4913 | No |
| 105 | PDE4B |  |  | 2788 | -0.333 | -0.4915 | No |
| 106 | PEX2 |  |  | 2792 | -0.334 | -0.4906 | No |
| 107 | ZCCHC24 |  |  | 2795 | -0.334 | -0.4895 | No |
| 108 | BMPR1A |  |  | 2800 | -0.335 | -0.4889 | No |
| 109 | METTL7A |  |  | 2818 | -0.339 | -0.4917 | No |
| 110 | IGFBP5 |  |  | 2820 | -0.339 | -0.4903 | No |
| 111 | SIRPA |  |  | 2823 | -0.340 | -0.4892 | No |
| 112 | IRF2 |  |  | 2830 | -0.341 | -0.4891 | No |
| 113 | FAM3C |  |  | 2850 | -0.344 | -0.4924 | No |
| 114 | C3orf70 |  |  | 2851 | -0.345 | -0.4907 | No |
| 115 | S100A13 |  |  | 2858 | -0.346 | -0.4906 | No |
| 116 | TTC14 |  |  | 2867 | -0.347 | -0.4910 | No |
| 117 | PTPN13 |  |  | 2871 | -0.348 | -0.4901 | No |
| 118 | ST3GAL5 |  |  | 2910 | -0.358 | -0.4983 | No |
| 119 | CTNND2 |  |  | 2919 | -0.359 | -0.4987 | No |
| 120 | PCDH17 |  |  | 2924 | -0.360 | -0.4980 | No |
| 121 | SLITRK2 |  |  | 2970 | -0.368 | -0.5080 | No |
| 122 | SNX3 |  |  | 2987 | -0.370 | -0.5104 | No |
| 123 | CPE |  |  | 2994 | -0.371 | -0.5102 | No |
| 124 | ADCY2 |  |  | 3005 | -0.374 | -0.5109 | No |
| 125 | ALDH6A1 |  |  | 3027 | -0.379 | -0.5146 | No |
| 126 | HIPK2 |  |  | 3029 | -0.379 | -0.5130 | No |
| 127 | PHYHIPL |  |  | 3049 | -0.385 | -0.5161 | No |
| 128 | CSGALNACT1 |  |  | 3052 | -0.386 | -0.5148 | No |
| 129 | SPRED1 |  |  | 3054 | -0.387 | -0.5131 | No |
| 130 | PAX6 |  |  | 3055 | -0.387 | -0.5112 | No |
| 131 | TNK2 |  |  | 3057 | -0.387 | -0.5096 | No |
| 132 | LAPTM4B |  |  | 3068 | -0.389 | -0.5103 | No |
| 133 | CASC4 |  |  | 3070 | -0.390 | -0.5087 | No |
| 134 | ABAT |  |  | 3075 | -0.391 | -0.5078 | No |
| 135 | ACOX1 |  |  | 3121 | -0.404 | -0.5177 | No |
| 136 | CREM |  |  | 3122 | -0.404 | -0.5157 | No |
| 137 | SAMD8 |  |  | 3123 | -0.404 | -0.5137 | No |
| 138 | BMP7 |  |  | 3127 | -0.407 | -0.5125 | No |
| 139 | HEY1 |  |  | 3130 | -0.408 | -0.5111 | No |
| 140 | GOLIM4 |  |  | 3154 | -0.414 | -0.5151 | No |
| 141 | NDUFB5 |  |  | 3158 | -0.415 | -0.5138 | No |
| 142 | PNISR |  |  | 3160 | -0.416 | -0.5121 | No |
| 143 | BDH2 |  |  | 3162 | -0.418 | -0.5103 | No |
| 144 | PLEKHG1 |  |  | 3167 | -0.419 | -0.5093 | No |
| 145 | TMED10 |  |  | 3179 | -0.423 | -0.5101 | No |
| 146 | P2RY1 |  |  | 3189 | -0.426 | -0.5104 | No |
| 147 | CD81 |  |  | 3196 | -0.427 | -0.5099 | No |
| 148 | TOMM7 |  |  | 3198 | -0.428 | -0.5080 | No |
| 149 | NOTCH1 |  |  | 3202 | -0.429 | -0.5067 | No |
| 150 | PON2 |  |  | 3216 | -0.432 | -0.5080 | No |
| 151 | SOX9 |  |  | 3230 | -0.435 | -0.5093 | No |
| 152 | NPAS3 |  |  | 3233 | -0.437 | -0.5077 | No |
| 153 | FYN |  |  | 3262 | -0.444 | -0.5129 | No |
| 154 | ITGA6 |  |  | 3315 | -0.461 | -0.5243 | No |
| 155 | S100A16 |  |  | 3338 | -0.472 | -0.5278 | No |
| 156 | CHP1 |  |  | 3354 | -0.479 | -0.5294 | No |
| 157 | RHOJ |  |  | 3375 | -0.488 | -0.5322 | Yes |
| 158 | ZMAT3 |  |  | 3380 | -0.490 | -0.5309 | Yes |
| 159 | PRNP |  |  | 3383 | -0.491 | -0.5290 | Yes |
| 160 | PTPRA |  |  | 3392 | -0.494 | -0.5287 | Yes |
| 161 | TMEM106B |  |  | 3402 | -0.499 | -0.5286 | Yes |
| 162 | TRIL |  |  | 3411 | -0.503 | -0.5283 | Yes |
| 163 | GAS1 |  |  | 3415 | -0.506 | -0.5266 | Yes |
| 164 | APC |  |  | 3422 | -0.510 | -0.5257 | Yes |
| 165 | RFTN2 |  |  | 3427 | -0.511 | -0.5242 | Yes |
| 166 | CACNB2 |  |  | 3452 | -0.521 | -0.5280 | Yes |
| 167 | SEMA6A |  |  | 3455 | -0.521 | -0.5259 | Yes |
| 168 | QKI |  |  | 3467 | -0.526 | -0.5263 | Yes |
| 169 | JAM2 |  |  | 3477 | -0.532 | -0.5260 | Yes |
| 170 | STAT3 |  |  | 3488 | -0.537 | -0.5260 | Yes |
| 171 | WSB1 |  |  | 3499 | -0.541 | -0.5260 | Yes |
| 172 | FABP5 |  |  | 3513 | -0.549 | -0.5267 | Yes |
| 173 | ITM2C |  |  | 3518 | -0.552 | -0.5251 | Yes |
| 174 | ARHGEF6 |  |  | 3530 | -0.560 | -0.5252 | Yes |
| 175 | VEGFA |  |  | 3532 | -0.560 | -0.5228 | Yes |
| 176 | AASS |  |  | 3539 | -0.564 | -0.5216 | Yes |
| 177 | OLIG1 |  |  | 3551 | -0.571 | -0.5217 | Yes |
| 178 | CD9 |  |  | 3567 | -0.581 | -0.5228 | Yes |
| 179 | ZEB1 |  |  | 3589 | -0.598 | -0.5254 | Yes |
| 180 | DBI |  |  | 3597 | -0.601 | -0.5243 | Yes |
| 181 | DNASE2 |  |  | 3598 | -0.603 | -0.5213 | Yes |
| 182 | BBX |  |  | 3600 | -0.605 | -0.5186 | Yes |
| 183 | SPATA6 |  |  | 3606 | -0.608 | -0.5170 | Yes |
| 184 | AIF1L |  |  | 3621 | -0.617 | -0.5176 | Yes |
| 185 | BAALC |  |  | 3628 | -0.625 | -0.5162 | Yes |
| 186 | PBXIP1 |  |  | 3635 | -0.633 | -0.5146 | Yes |
| 187 | CDCA7L |  |  | 3642 | -0.637 | -0.5131 | Yes |
| 188 | MEIS1 |  |  | 3651 | -0.648 | -0.5120 | Yes |
| 189 | MLC1 |  |  | 3658 | -0.652 | -0.5104 | Yes |
| 190 | SAT1 |  |  | 3659 | -0.653 | -0.5072 | Yes |
| 191 | DAG1 |  |  | 3662 | -0.654 | -0.5046 | Yes |
| 192 | ID2 |  |  | 3664 | -0.654 | -0.5016 | Yes |
| 193 | LITAF |  |  | 3670 | -0.660 | -0.4997 | Yes |
| 194 | OLFM2 |  |  | 3690 | -0.672 | -0.5014 | Yes |
| 195 | RNF13 |  |  | 3696 | -0.681 | -0.4994 | Yes |
| 196 | STON2 |  |  | 3702 | -0.689 | -0.4974 | Yes |
| 197 | ZMYM2 |  |  | 3703 | -0.689 | -0.4940 | Yes |
| 198 | TIMP2 |  |  | 3705 | -0.691 | -0.4909 | Yes |
| 199 | PCDH9 |  |  | 3707 | -0.691 | -0.4878 | Yes |
| 200 | PLEKHH2 |  |  | 3711 | -0.693 | -0.4852 | Yes |
| 201 | VEGFB |  |  | 3726 | -0.705 | -0.4854 | Yes |
| 202 | TGIF1 |  |  | 3728 | -0.707 | -0.4822 | Yes |
| 203 | FAT1 |  |  | 3730 | -0.708 | -0.4790 | Yes |
| 204 | CAMK2D |  |  | 3737 | -0.714 | -0.4771 | Yes |
| 205 | HTRA1 |  |  | 3741 | -0.719 | -0.4744 | Yes |
| 206 | GNG7 |  |  | 3746 | -0.724 | -0.4719 | Yes |
| 207 | CTNNB1 |  |  | 3753 | -0.731 | -0.4699 | Yes |
| 208 | RAB31 |  |  | 3757 | -0.737 | -0.4671 | Yes |
| 209 | NCAN |  |  | 3763 | -0.748 | -0.4647 | Yes |
| 210 | SCD5 |  |  | 3765 | -0.751 | -0.4613 | Yes |
| 211 | TGFB2 |  |  | 3774 | -0.766 | -0.4597 | Yes |
| 212 | PRCP |  |  | 3775 | -0.766 | -0.4559 | Yes |
| 213 | SALL3 |  |  | 3778 | -0.768 | -0.4527 | Yes |
| 214 | ARHGAP12 |  |  | 3784 | -0.773 | -0.4502 | Yes |
| 215 | MAML2 |  |  | 3793 | -0.783 | -0.4485 | Yes |
| 216 | SPECC1 |  |  | 3801 | -0.790 | -0.4465 | Yes |
| 217 | NACC2 |  |  | 3803 | -0.792 | -0.4429 | Yes |
| 218 | TRPS1 |  |  | 3804 | -0.793 | -0.4390 | Yes |
| 219 | TTYH1 |  |  | 3807 | -0.795 | -0.4356 | Yes |
| 220 | SOX8 |  |  | 3809 | -0.797 | -0.4320 | Yes |
| 221 | WSCD1 |  |  | 3817 | -0.803 | -0.4299 | Yes |
| 222 | NLGN4X |  |  | 3823 | -0.810 | -0.4273 | Yes |
| 223 | FJX1 |  |  | 3824 | -0.813 | -0.4233 | Yes |
| 224 | MAGI1 |  |  | 3827 | -0.815 | -0.4199 | Yes |
| 225 | NLGN1 |  |  | 3836 | -0.828 | -0.4179 | Yes |
| 226 | RHOBTB3 |  |  | 3838 | -0.829 | -0.4141 | Yes |
| 227 | IDH1 |  |  | 3842 | -0.832 | -0.4108 | Yes |
| 228 | LYPD1 |  |  | 3844 | -0.834 | -0.4070 | Yes |
| 229 | LDLRAD3 |  |  | 3857 | -0.849 | -0.4060 | Yes |
| 230 | PSAP |  |  | 3882 | -0.907 | -0.4079 | Yes |
| 231 | SRI |  |  | 3885 | -0.909 | -0.4040 | Yes |
| 232 | DSEL |  |  | 3897 | -0.940 | -0.4023 | Yes |
| 233 | DUSP6 |  |  | 3899 | -0.946 | -0.3979 | Yes |
| 234 | DDR1 |  |  | 3903 | -0.948 | -0.3941 | Yes |
| 235 | FOS |  |  | 3911 | -0.966 | -0.3912 | Yes |
| 236 | LRRN1 |  |  | 3913 | -0.970 | -0.3867 | Yes |
| 237 | SEL1L3 |  |  | 3919 | -1.001 | -0.3831 | Yes |
| 238 | SEMA5A |  |  | 3921 | -1.006 | -0.3785 | Yes |
| 239 | CHPT1 |  |  | 3923 | -1.006 | -0.3738 | Yes |
| 240 | STK17A |  |  | 3925 | -1.012 | -0.3691 | Yes |
| 241 | BCHE |  |  | 3926 | -1.012 | -0.3642 | Yes |
| 242 | TRIM9 |  |  | 3930 | -1.016 | -0.3600 | Yes |
| 243 | ETV1 |  |  | 3934 | -1.026 | -0.3558 | Yes |
| 244 | GPRC5B |  |  | 3937 | -1.032 | -0.3513 | Yes |
| 245 | SLC1A3 |  |  | 3942 | -1.040 | -0.3473 | Yes |
| 246 | PCDHGC3 |  |  | 3943 | -1.040 | -0.3422 | Yes |
| 247 | TRIB2 |  |  | 3944 | -1.041 | -0.3371 | Yes |
| 248 | DCLK2 |  |  | 3945 | -1.043 | -0.3320 | Yes |
| 249 | ITPR2 |  |  | 3946 | -1.044 | -0.3269 | Yes |
| 250 | RNF180 |  |  | 3949 | -1.052 | -0.3223 | Yes |
| 251 | SOX2 |  |  | 3953 | -1.069 | -0.3178 | Yes |
| 252 | PTPRG |  |  | 3958 | -1.083 | -0.3136 | Yes |
| 253 | ITGB8 |  |  | 3962 | -1.093 | -0.3090 | Yes |
| 254 | ETV5 |  |  | 3971 | -1.116 | -0.3057 | Yes |
| 255 | NEAT1 |  |  | 3976 | -1.142 | -0.3012 | Yes |
| 256 | MMP16 |  |  | 3983 | -1.171 | -0.2970 | Yes |
| 257 | RASSF2 |  |  | 3986 | -1.174 | -0.2918 | Yes |
| 258 | NPNT |  |  | 3989 | -1.189 | -0.2865 | Yes |
| 259 | MYO10 |  |  | 3990 | -1.193 | -0.2807 | Yes |
| 260 | ITM2B |  |  | 3995 | -1.225 | -0.2757 | Yes |
| 261 | FGFBP3 |  |  | 3999 | -1.237 | -0.2705 | Yes |
| 262 | GPM6A |  |  | 4002 | -1.248 | -0.2649 | Yes |
| 263 | SESN3 |  |  | 4003 | -1.260 | -0.2588 | Yes |
| 264 | NOVA1 |  |  | 4008 | -1.312 | -0.2534 | Yes |
| 265 | CLSTN1 |  |  | 4020 | -1.391 | -0.2495 | Yes |
| 266 | ZBTB20 |  |  | 4024 | -1.407 | -0.2434 | Yes |
| 267 | LSAMP |  |  | 4028 | -1.442 | -0.2371 | Yes |
| 268 | MAP3K1 |  |  | 4032 | -1.477 | -0.2307 | Yes |
| 269 | SLC35F1 |  |  | 4035 | -1.515 | -0.2238 | Yes |
| 270 | FAM181B |  |  | 4037 | -1.542 | -0.2166 | Yes |
| 271 | TNC |  |  | 4039 | -1.549 | -0.2092 | Yes |
| 272 | SLC1A2 |  |  | 4040 | -1.555 | -0.2017 | Yes |
| 273 | SYT11 |  |  | 4047 | -1.617 | -0.1953 | Yes |
| 274 | CADM4 |  |  | 4053 | -1.687 | -0.1884 | Yes |
| 275 | LIMA1 |  |  | 4056 | -1.716 | -0.1805 | Yes |
| 276 | LRRN3 |  |  | 4064 | -1.810 | -0.1735 | Yes |
| 277 | CXCL14 |  |  | 4066 | -1.885 | -0.1646 | Yes |
| 278 | CLU |  |  | 4069 | -1.908 | -0.1558 | Yes |
| 279 | SCRG1 |  |  | 4070 | -2.021 | -0.1459 | Yes |
| 280 | PTN |  |  | 4077 | -2.187 | -0.1368 | Yes |
| 281 | ID4 |  |  | 4079 | -2.308 | -0.1258 | Yes |
| 282 | GFAP |  |  | 4080 | -2.343 | -0.1143 | Yes |
| 283 | S100B |  |  | 4081 | -2.383 | -0.1027 | Yes |
| 284 | PMP2 |  |  | 4082 | -2.398 | -0.0910 | Yes |
| 285 | GPM6B |  |  | 4083 | -2.435 | -0.0791 | Yes |
| 286 | C1orf61 |  |  | 4084 | -2.474 | -0.0670 | Yes |
| 287 | IGFBP2 |  |  | 4094 | -3.345 | -0.0530 | Yes |
| 288 | FABP7 |  |  | 4095 | -3.351 | -0.0366 | Yes |
| 289 | PDGFRA |  |  | 4096 | -3.521 | -0.0194 | Yes |
| 290 | PTPRZ1 |  |  | 4097 | -4.027 | 0.0003 | Yes |
Table: GSEA details [plain text format]

  

Fig 2: RICHARDS\_NOWAKOWSKI\_SCIENCE\_2017\_ASTROCYTE\_UPREG: Random ES distribution      
 Gene set null distribution of ES for **RICHARDS\_NOWAKOWSKI\_SCIENCE\_2017\_ASTROCYTE\_UPREG**
